# Deciphering Genetic Susceptibility to Tuberculous Meningitis

**DOI:** 10.3389/fneur.2022.820168

**Published:** 2022-03-25

**Authors:** Haiko Schurz, Brigitte Glanzmann, Nicholas Bowker, Ronald van Toorn, Regan Solomons, Johan Schoeman, Paul D. van Helden, Craig J. Kinnear, Eileen G. Hoal, Marlo Möller

**Affiliations:** ^1^DSI-NRF Centre of Excellence for Biomedical Tuberculosis Research, South African Medical Research Council Centre for Tuberculosis Research, Division of Molecular Biology and Human Genetics, Faculty of Medicine and Health Sciences, Stellenbosch University, Cape Town, South Africa; ^2^SAMRC Genomics Centre, Cape Town, South Africa; ^3^Department of Paediatrics and Child Health, Faculty of Medicine and Health Sciences, Stellenbosch University, Stellenbosch, South Africa; ^4^Centre for Bioinformatics and Computational Biology, Stellenbosch University, Stellenbosch, South Africa

**Keywords:** tuberculous meningitis, pulmonary tuberculosis, exome sequencing, microarray, genome-wide association study

## Abstract

Tuberculous meningitis (TBM) is the most severe form of extrapulmonary tuberculosis (TB) that arises when a caseating meningeal granuloma discharges its contents into the subarachnoid space. It accounts for ~1% of all disease caused by *Mycobacterium tuberculosis* and the age of peak incidence is from 2–4 years. The exact pathogenesis of TBM is still not fully understood and the mechanism(s) by which the bacilli initially invade the blood-brain-barrier are still to be elucidated. This study investigated the involvement of the host genome in TBM susceptibility, by considering common variants (minor allele frequency (MAF) >5%) using microarray genotyping and rare variants (MAF <1%) via exome sequencing. A total of 123 TBM cases, 400 pulmonary TB (pTB) cases and 477 healthy controls were genotyped on the MEGA array. A genome-wide association study (GWAS) comparing 114 TBM cases to 395 healthy controls showed no association with TBM susceptibility. A second analysis comparing 114 TBM cases to 382 pTB cases was conducted to investigate variants associated with different TB phenotypes. No significant associations were found with progression from pTB to TBM. Ten TBM cases and 10 healthy controls were exome sequenced. Gene set association tests SKAT-O and SKAT Common Rare were used to assess the association of rare SNPs and the cumulative effect of both common and rare SNPs with susceptibility to TBM, respectively. Ingenuity Pathway Analysis (IPA) of the top-hits of the SKAT-O analysis showed that *NOD2* and *CYP4F2* are both important in TBM pathogenesis and highlighted these as targets for future study. For the SKAT Common Rare analysis Centriolar Coiled-Coil Protein 110 (*CCP110)* was nominally associated (*p* = 5.89x10^−6^) with TBM susceptibility. In addition, several top-hit genes ascribed to the development of the central nervous system (CNS) and innate immune system regulation were identified. Exome sequencing and GWAS of our TBM cohort has identified a single previously undescribed association of *CCP110* with TBM susceptibility. These results advance our understanding of TBM in terms of both variants and genes that influence susceptibility. In addition, several candidate genes involved in innate immunity have been identified for further genotypic and functional investigation.

## Introduction

Tuberculous meningitis (TBM) is the most severe form of extrapulmonary tuberculosis (TB) that arises when a caseating meningeal granuloma (Rich focus) discharges its contents into the subarachnoid space. It occurs most often in young children between the ages of 2 and 4 years (1, 2). This devastating form of TB generally occurs within a few of months following primary infection (3). Although the infectious agent, *Mycobacterium tuberculosis* (*M.tb*), is the same for TBM and pulmonary TB (pTB), the exact pathogenesis of TBM is still not fully understood (2). The dissemination is likely haematogenous from blood vessels of the sub-arachnoid space, leading to detrimental inflammation of the surrounding meningeal tissues. TBM represents only 1% of the total TB burden worldwide, but is the TB phenotype with the highest morbidity and mortality–an estimated 50% of patients do not survive or suffer neurological sequelae and complications (4). In the Western Cape of South Africa, the incidence rate was 31.5/100 000 in 1992, measured in children under 1 year old (5). The high number of South Africans living with HIV, 7.8 million in 2020 (6), has a major influence on the high prevalence of both TBM and TB in general. The mortality rate of HIV and TBM coinfection has been reported to be at 60% in a Vietnamese population (7). HIV coinfection was not found to alter the clinical presentation of the disease although it increased the likelihood for presentation with additional forms of extrapulmonary TB and decreased the survival rate of patients (8).

Host genetic susceptibility is another known risk factor for active TB, as is evident from the results of twin studies–monozygotic twins have higher concordance for the disease than dizygotic twins (9–11). Human genetic susceptibility to active TB has since been investigated in multiple genome-wide association studies (GWAS) and candidate gene associations studies in cohorts of various ethnic backgrounds (12–26). While results are rarely concordant between populations, GWAS have identified significantly associated variants and meta-analysis of toll-like receptor (*TLR*) candidate gene associations have identified significantly associated variants across multiple populations (27–29). The feasibility of using next generation sequencing technologies was demonstrated by an exome sequencing study of five participants (three with a history of active TB and two with a positive TB skin test indicative of latent *M.tb* infection) (30). This small-scale pilot study presented several potential candidate genes for further investigation. An association study making use of exome sequencing data from 119 pulmonary Indian TB cases and household controls reported novel associations with variants in the Sialic Acid Binding Ig Like Lectin 15 (*SIGLEC15)* and Major Histocompatibility Complex, Class II, DR Alpha (*HLA-DRA)* genes (31). Exome sequencing data also allowed the identification of a missense tyrosine kinase 2 (*TYK2)* gene variant, P1104A, which selectively disrupts the induction of interferon-γ by interleukin-23 and is a common monogenic etiology of tuberculosis in non-European countries where TB is endemic (32). This same variant may account for ~1% of TB in Europeans (33).

Genetic susceptibility to TBM has been researched less intensively and with older genotyping technologies, but these studies using TBM cohorts have identified associations with specific candidate genes and variants first associated with pTB. The C allele of g.597T>C in the toll-like receptor 2 (*TLR2*) gene was associated with increased susceptibility to TBM in a Vietnamese cohort when TBM, miliary TB and severe neurological symptoms were concurrent [*p* = 0.0002 (OR = 5.70, 95% CI: 1.81–18.0)] (34). Polymorphisms in *TLR2* may alter the heterodimerisation capability of TLR2 with either TLR1 or TLR6 and thus affect the capacity for ligand recognition. Further studies have linked SNPs in genes such as interferon-γ (*IFN-*γ), interleukin-4 (*IL-4*) and toll-interleukin 1 receptor domain containing adaptor protein (*TIRAP*) with increased susceptibility to TBM (35–38). While this extrapolated approach based on pTB susceptibility provides valuable information, it is likely to miss TBM-specific associations, such as that of leukotriene A4 hydrolase (LTA4H) which regulates the balance between pro- and anti-inflammatory cytokines (39). The homozygous CC genotype of the *LTA4H* promoter rs17525495 variant leads to an anti-inflammatory phenotype which lowers tumor necrosis factor alpha (TNF-α) to detrimental levels. Conversely TT genotypes lead to a hyper-inflammatory phenotype and high TNF-α concentrations. Heterozygous genotypes maintain the delicate balance between the pro- and anti-inflammatory responses and protects against pTB and TBM in a Vietnamese population (40, 41). Heterozygote advantage is uncommon and suggests that dysregulation of the inflammatory response is linked to TBM susceptibility (42). The rs17525496 variant was also associated with survival from TBM in Vietnam, but not in Indonesia (43–45).

The present study made use of a genome-wide approach and exome sequencing in a South African population to determine if single nucleotide polymorphisms (SNPs) are associated with increased susceptibility to TBM at a genome-wide significant level. Here we report on GWAS between TBM cases and healthy controls and between TBM cases and pTB, using pTB cases and controls from our previous GWAS (19). This study represents the first GWAS using a TBM cohort from South Africa and includes exome sequencing data generated for this extreme TB phenotype.

## Materials and Methods

### Setting

The Western Cape Province has one of the highest TB incidence rates in the world, 681 per 100 000, as reported in 2015 (46). This province also has one of the highest TBM incidence rates in the world, 31.5 per 100 000 in children younger than 1 year (47). Population genetics analyses have revealed a complex five-way admixture in individuals from the Western Cape (SAC) which includes contributions from European, East Asian, South Asian, Bantu-speaking African and Khoe-San populations (48). To account for the possible effects of multiple contributing ancestral populations, it is necessary to correct for population stratification in our genetic analyses (49).

### Study Participants

#### TBM Study Participants

HIV-negative TBM patient samples were collected and stored as part of ongoing recruitment at Tygerberg Children's Hospital, Cape Town, South Africa since 1991. Approximately 3 TBM cases are diagnosed per month. Diagnosis is classified into two sub-categories “Definite TBM” and “Probable TBM. Definite TBM was diagnosed in cases where AFB were seen on CSF microscopy, positive CSF *Mycobacterium tuberculosis* culture or GeneXpert. Probable TBM was diagnosed when a score of 12 or more was achieved using the uniform TBM research case definition (50). Population and language information was collected for each individual recruited for the study. The TBM participants self-identified as SAC (*n* = 96) or Xhosa (*n* = 30) individuals. Sample characteristics, including sex, age and ancestry proportions, are shown in Table 1.

**Table 1:** Sample characteristics including sex, age, and ancestry proportions per study arm.

		**Study**		
	**Exome sequencing^a^**	**GWAS TBM vs. Controls**	**GWAS TBM vs. pTB**
Cases	Number of cases	10	114	114
	Nr Males (prop)	5 (0.50)	59 (0.52)	59 (0.52)
	Age (mean ± SD)	5.1 ± 3.93	5.24 ± 4.86	5.24 ± 4.86
	African Khoe-San [IQR]	0.27 [0.24–0.35]	0.29 [0.22–0.38]	0.29 [0.22–0.38]
	African non-San [IQR]	0.42 [0.31–0.74]	0.36 [0.24–0.65]	0.36 [0.24–0.65]
	European [IQR]	0.09 [1 x 10.5–0.14]	0.04 [1 x 10.5–0.10]	0.04 [1 x 10.5–0.10]
	South Asian [IQR]	0.08 [1 x 10.5–0.14]	0.19 [0.05–0.26]	0.19 [0.05–0.26]
Controls	Number of controls	10	395	382
	Nr Males (prop)	5 (0.50)	118 (0.30)	212 (0.55)
	Age (mean ± SD)	49.65 ± 12.79	30.88 ± 13.10	36.32 ± 11.04
	African Khoe-San [IQR]	0.31 [0.27–0.45]	0.25 [0.19–0.34]	0.31 [0.20–0.40]
	African non-San [IQR]	0.20 [0.15–0.24]	0.27 [0.19–0.38]	0.24 [0.15–0.36]
	European [IQR]	0.24 [0.22–0.29]	0.12 [0.05–0.20]	0.17 [0.12–0.22]
	South Asian [IQR]	0.11 [0.08–0.15]	0.25 [0.18–0.31]	0.15 [0.10–0.21]

a *The exome sequencing was exploratory and limited to 20 participants due to the budget available*.

*IQR, Interquartile range; SD, Standard deviation; Prop, Proportion; GWAS, Genome-Wide Association Study*.

*To avoid linear dependency in the data, the East Asian component was not added to the model*.

*P-values reflect the significance of the association of each factor with TB, adjusted for the other factors*.

#### Pulmonary TB Patients

Pulmonary TB patients (*n* = 382) included in the study self-identified as part of the SAC population and were recruited from two metropolitan areas of Cape Town. These areas were selected due to the high TB incidence (1 340 per 100 000) and low HIV prevalence (~2% of the population) at the time of sampling (51, 52). All study participants were HIV negative, unrelated, and over 18 years of age. Diagnosis of pTB was determined through bacteriological confirmation using either smear and/or culture methods for positivity, as described previously (53).

#### Healthy Control Individuals

Control samples (*n* = 395) were collected from the same metropolitan areas as the pTB cases and therefore share the same environmental and socio-economic circumstances. Controls were over the age of 18, HIV-negative and unrelated to one another and to the cases (19, 53). Additionally, control samples were defined as individuals who had never had a case of active TB in their lifetime, but are assumed to be latently infected, based on the fact that over 80% of individuals in the area over the age of 15 are tuberculin skin test (TST) positive, indicating significant exposure of control individuals to *M.tb* (54).

### Genotyping

DNA samples were submitted for genotyping at the Hussman Institute for Human Genomics (HIHG) (University of Miami, Florida, USA) using the Illumina Multi-Ethnic Genotyping Array (MEGA) platform (Illumina San Diego, CA, USA) (19). Considering the available sample size, we had 82% power at an alpha level of 0.05 to detect an association (additive model) with an odds ratio of 5, a minor allele frequency (MAF) of 0.01, and a disease prevalence of 1% as per the CaTS power calculator (55).

#### Array Data Quality Control

PLINK v1.07 (56) was used for quality control (QC) of the array data. First duplicated and improperly mapped (chromosome 0) SNPs were removed from the data. Next variant QC was performed by filtering for Hardy Weinberg equilibrium (controls only, *p* < 0.05), genotype missingness (>10%) and MAF (<5%). Following this sample QC was conducted by filtering for individual missingness (>10%), cryptic relatedness (cut-off = >0.185) and sex concordance. Finally, the sex chromosomes were removed as only autosomal regions were of interest for this study.

#### Exome Sequencing

The input genomic DNA was sonicated to fragment the DNA to a size of 150 bp and quality and size assessment performed using the Agilent Bioanalyser 2100 and DNA 1000 chip and reagent kit (Agilent Technologies Santa Clara, CA, USA). Targeted enrichment for exonic sites was done using the Nextera XT enrichment kit (Illumina San Diego, CA, USA) which targeted >20 000 genes between 40 and 60 x read depth. A total of 6 μg of DNA was used for library preparation for the Illumina Nextera XT library (Illumina San Diego, CA, USA). Paired-end sequencing was performed on the Illumina HiSeq 2500 (San Diego, CA, USA) at the Christian-Albrechts University of Kiel (CAU sequencing Kiel, Germany). Sequencing data was received in.fastq format and data quality was analyzed using FastQC v0.11.5. Burrows-Wheeler Aligner (BWA)-MEM (version 0.7.17), with default parameters, was used to align all sequencing reads to the human reference genome GRCh37p13 (https://www.ncbi.nlm.nih.gov/assembly/GCF_000001405.13/). The quality of the aligned reads was assessed using SAMtools (version 1.9). Duplicate reads were removed using Picard v2.2.1 (http://picard.sourceforge.net/). Variants were called using HaplotypeCaller producing a single variant called format (VCF) file for all samples. Variant annotation was done using wANNOVAR software. The annotated VCF file was filtered to prioritize rare SNPs with a MAF of 0.01 and below in both the 1000 Genomes Project (57) and Exome Sequencing Project (ESP6500si) (58). Final MAF filtration was done using frequency information from the Exome Aggregation Consortium (ExAC) (59). Non-synonymous variants, frameshift SNPs, SNPs that induced stop codon gains or losses, splice-site SNPs and insertions and deletions were retained during filtration. Filtration for the retention of conserved sites was performed based on PhyloP (60) and GERP++ scores (61) and SNPs with negative scores from both conservation annotators were removed.

### Admixture Inference

The SAC population is a five-way admixed population with ancestral contributions from Bantu-speaking African populations, Khoe-San, Europeans and South and East Asians, while Xhosa individuals also display admixture. To avoid confounding during association testing the ancestral components are included as covariates (53). Admixture was inferred on the autosomes as described previously (19). Briefly the ADMIXTURE (v1.3) software was used in combination with reference genotyping data of the five ancestral populations (62). The reference populations used to infer ancestry were European (CEU) and South Asian (Gujarati Indians in Houston, Texas, and Pathan of Punjab) extracted from the 1000 Genomes Phase 3 data (63), East Asian (Han Chinese in Beijing, China), African (Luhya in Webuye, Kenya, Bantu-speaking African, Yoruba from Nigeria) and San (Nama/Khomani) (64, 65). Four ancestral components (African, San, European and South Asian) were included as covariates in the logistic regression association testing with the smallest component (East Asian) excluded to avoid complete separation of the data (66).

### Statistical Analysis

#### Genome-Wide Association Analysis

Association testing for both TBM vs. healthy controls and TBM vs. pTB cases was done using PLINK (v1.07) to implement an additive logistic regression model (56). Both analyses adjusted for confounding effects by including sex and the four main ancestral components as covariates. Age was not included as a covariate due to the large difference between the ages of TBM cases compared to both healthy controls and pTB cases. The genome-wide significance threshold for the GWAS analysis was set to 5.0 x 10^−8^ (67). The variant effect predictor (VEP) accessible via the Ensembl genome browser was used determine the genes and regulatory regions affected by changes to the nucleotide sequence (68). Additionally, transcription factor binding site annotations were accessed using the UCSC Table Browser facility (69) and functional annotation of the GWAS summary statistics was done using FUMA (70, 71).

#### FUMA Functional Annotation

To further analyze the GWAS results, summary statistics from both analyses (TBM vs. healthy control and TBM vs. pTB) were functionally annotated using the freely available online tool FUMA (70, 71). GWAS summary statistics were used for a gene-based and gene set analysis to identify potential genes of interest. Potential candidate genes were then further investigated by inferring differential expression (DE) and tissue specific expression as well as cis eQTL markers, based on publicly available reference databases. A lenient genome-wide significance threshold of 1e^−5^ was used for functional enrichment annotation.

#### SKAT-O and SKAT Common Rare Analysis

SKAT-O analysis was performed to assess the rare-variant burden across all genes containing rare SNPs identified using exome sequencing. Using the R free programming environment, with the SKAT R package, gene set input files (SKAT-specific SSD and SSD.Info files) were created by grouping SNPs into genes, based on the SKAT provided gene set reference file (72, 73).

Prior to running SKAT-O, a null model was created to aid in the determination of p-values in the analysis. The null model was constructed to incorporate dichotomous phenotypes of TBM patients and healthy controls regressed against the study covariates. A single covariate of gender was incorporated into the model. During the construction of the null model, only sex was included as a covariate, as including any of the others in conjunction with sex resulted in complete separation of the data. The test was conducted under the small-sample size kurtosis adjustment, implemented by the SKAT package, as the sample size was under 2 000 individuals. Optimisation of the test statistics was modulated by the program and tested across the default distribution of ρ (0, 0.1^2^, 0.2^2^, 0.3^2^, 0.4^2^, 0.5^2^, 0.5, and 1) to minimize the *p*-value produced during analysis, which was then used as the test *p*-value. *P*-values were Bonferroni adjusted for multiple testing and the significance threshold was set to *p* ≤ 0.05.

**Figure 1:**
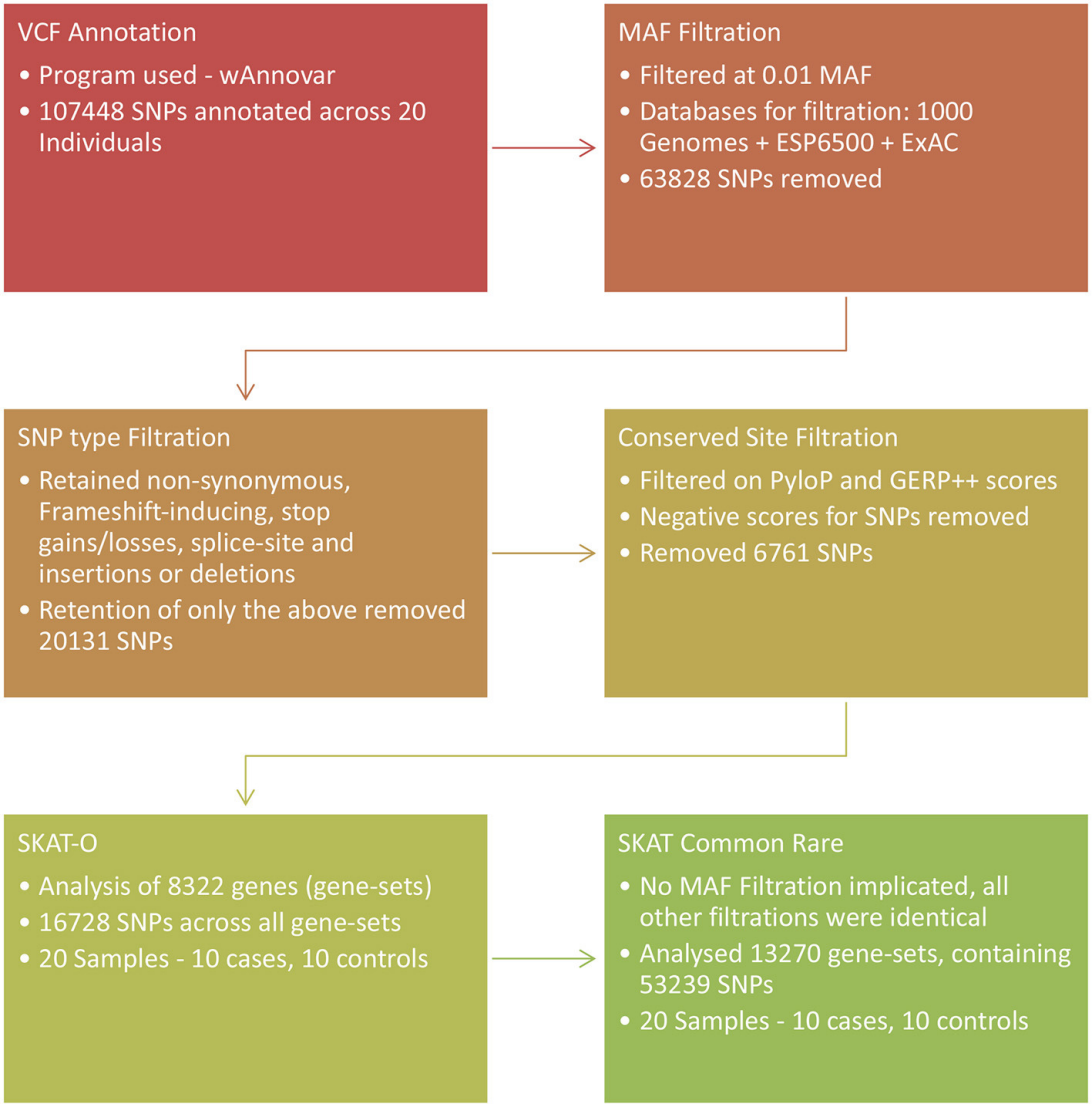
Overview of the SNP prioritization procedures followed and association analyses used in the exome sequencing arm of the study. SKAT, Sequence Kernel Association Test.

To test the combined effect association of both common and rare SNPs in the analysis, the common/rare SNP association test as part of the SKAT R package was used (72, 73). Gene set files included common variants for this analysis, and the Null model was created as for the SKAT-O analysis and the common rare analysis was implemented using the R package, SKAT, Common Rare function (72–74). The common and rare variants were tested in two groups separated based on an adaptive MAF value as a function of the total sample size. The *p*-values of the two groups were then combined and weights assigned according to MAF on a per SNP basis. Test results were corrected for multiple testing at a *p*-value significance cut-off of 0.05. An outline of the SNP filtration and prioritization procedures for the two SKAT analysis is shown in Figure 1.

#### Ingenuity Pathway Analysis of SKAT Results

Functional assignment and pathway analysis of the association results was done to prioritize genes of interest from the SKAT Common-Rare and SKAT-O analyses. Association results were filtered for the retention of unadjusted *p*-values below a threshold of 0.01, allowing for sufficient genes for input. Using IPA core analysis, the top hits from both SKAT association tests were assessed independently (75). Analysis included overrepresentation analysis of pathways assigned to the input genes, network construction between gene products and genes, functional assignment, disease associations and assessment of shared regulatory molecules of the input genes. Analysis filtration parameters were defined to draw information from the Ingenuity Knowledge Base (genes only). Network construction was not constrained as exploration into related genes was desired, thus direct, and indirect relationships were used. Filtration parameters for results pertaining only to *Homo sapiens* were applied to exclude any gene functions annotated through homology from other species. Additionally, filtration was applied to include only experimentally confirmed interactions, thus providing maximum confidence in the results.

## Results

### Genome-Wide Association Studies

Following QC of the MEGA array data 1 465 892 SNPs in 116 TBM cases, 382 pTB cases and 430 healthy controls remained for downstream analysis.

#### TBM Cases vs. Healthy Controls

No significant associations with TBM susceptibility were found (Supplementary Table 1). The lowest *p*-value was that of rs77857429 [*p* = 5.68x10^−6^ (OR = 4.28; 95% CI: 2.28–8.02)], an intronic SNP located in the Glucosylceramidase Beta 3 (*GBA3*) gene. Four of the SNPs in the top hits are in transcription factor binding sites (Supplementary Table 2). None of the SNPs were exonic. The Plexin B2 gene (*PLXNB2*) encodes several transcripts and, depending on the transcript, the rs1127000 SNP is either a splice site variant or a synonymous SNP. However, using VEP, rs1127000 was predicted to be of low effect when all transcripts were considered (68). Variants in genes previously associated with TB and TBM were nominally associated with disease (*p* < 0.05), but no associations reached genome wide significance (Supplementary Table 3).

#### TBM Cases vs. pTB Cases

No significant associations were identified when comparing the pTB and TBM cases (Supplementary Table 4, significance threshold *p* = 5.0x10^−8^). The SNP with the lowest *p-*value was rs2679308 (*p* = 5.11 x 10^−6^), located in an intron in *LOC102724084*, an uncharacterised gene. The top hits were annotated for gene, SNP type and transcription factor binding site annotations (Supplementary Table 5). The rs2251220 SNP, in *KIAA1549*, induces a non-synonymous change from Serine to Leucine in the protein. VEP analysis of rs2251220 using SiFT and Polyphen plugins classified the SNP as tolerated and benign with scores of 0.1 and 0.053, respectively. The rs4309447 and rs2140779 SNPs are in transcription factor binding sites. The rs2140779 SNP was located in a transcription factor binding site upstream of the Glioma-Associated Oncogene Family Zinc Finger 2 (*GLI2*) gene and is bound by the Jun transcription factor. This suggests a possible role in both the cis- and trans-regulation of several genes. Comparing these TBM vs. PTB results with previously associated TB and TBM candidate genes also only identified nominal associations (Supplementary Table 6).

#### Functional Annotation of GWAS Results

**Figure 2:**
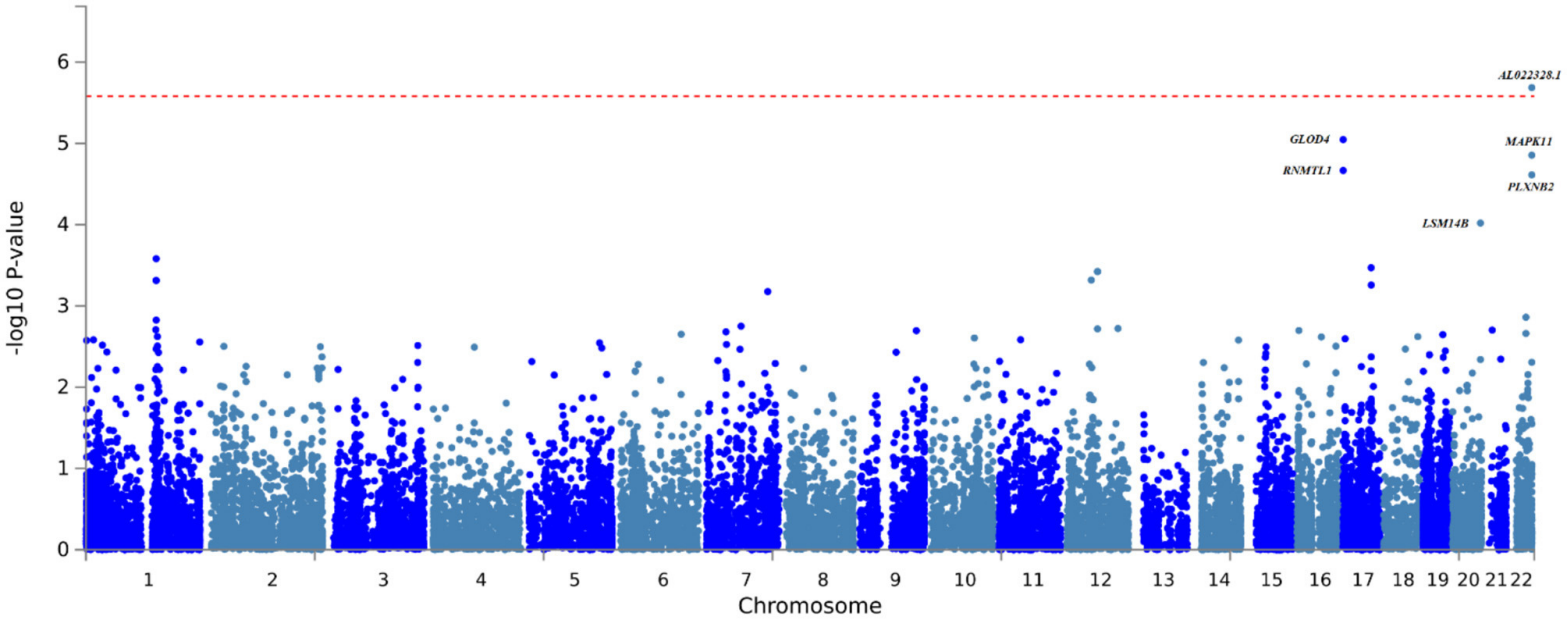
Gene-based association testing results obtained from the FUMA analysis of the TBM vs. healthy control GWAS summary statistics.

The FUMA gene-based and gene set analysis revealed significant associations for the TBM vs. healthy controls following Bonferroni correction for testing 19 108 genes (Figure 2, Supplementary Table 7). The long non-coding RNA (lncRNA) *AL022328.1* was significantly associated with disease (*p*-value: 2.0554e^−6^), however its role in TBM susceptibility is unclear and the functional impact of this lncRNA has not been fully established. While not reaching the Bonferroni corrected significance level among the most significant associations *MAPK11* (mitogen-activated protein kinase 11) is in linkage disequilibrium with *AL022328.1* and can thus be considered as a single locus (Supplementary Table 7 and Supplementary Figure 1). *MAPK11*, along with the next most significant association in the same genomic locus, *PLXNB2*, are strong candidates for TBM susceptibility as *MAPK* genes have previously been implicated in TB susceptibility and *PLXNB2* is involved in immune response regulation (76, 77). The glyoxalase domain containing 4 gene (*GLOD4)*, was also among the top hits, but its contribution to TBM susceptibility is not known. Gene set analysis compares the *p*-value distribution (from the gene-based associations) of genes belonging to certain pathways to each other to determine if any gene sets are significantly associated with TBM compared to all other gene sets. Genes involved in ADP ribosylation were significantly associated with TBM following Bonferroni correction (*p-*value: 1.26e^−4^). ADP ribosylation is a reversable protein post-translational modifier controlling major cellular and biological processes including cell proliferation and differentiation and immune responses and could impact TBM susceptibility (78). FUMA analysis did not reveal any further significant associations (DE, tissue specific expression, eQTL or enrichment) for both the TBM vs. healthy control and TBM vs. pTB analysis.

### Exome Sequencing Results

The depth of the exome sequencing ranged from 35 x to 82 x. The mean depth across all 20 individuals was 52 x. A total of 107 448 SNPs were called across the 20 study participants. SNP prioritization for the SKAT-O analysis removed 63 828 SNPs that were above a MAF of 1%. An additional 20 131 SNPs were removed during filtration for the inclusion of non-synonymous and frameshift inducing SNPs, stop gains/losses, splice-site SNPs and insertions and deletions. A further 6 761 SNPs were removed because of the conservation score, resulting in 16 728 SNPs for SKAT-O analysis. The SKAT Common Rare prioritization removed 35 589 SNPs to prioritize non-synonymous and frameshift inducing SNPs. A further 18 620 variants were removed based upon conservation score and 53 239 SNPs remained for SKAT Common Rare analysis.

SKAT-O analysis was performed on 8 322 gene-sets containing 16 728 SNPs using a dichotomous phenotype of TBM patients compared to healthy controls. None of the gene-sets were found to be associated after correcting for multiple testing using a Bonferroni significance level of *p* = 0.05. The gene-set with the lowest *p*-value was that of Zinc Finger Homeobox-3 (*ZFHX3*), *p* = 4.63 x 10^−4^.

SKAT Common Rare analysis was performed on 13 270 gene-sets and 53 239 SNPs, and a single significant association was identified by comparing TBM cases and controls after correcting for multiple testing. The Centriolar Coiled-Coil Protein 110kDa (*CCP110*) gene was found to be borderline associated with TBM (*p* = 5.89x10^−6^) after correcting for multiple testing, with the combined effects of 1 rare SNP and 2 common SNPs contributing to the association.

#### IPA Analysis of SKAT-O and SKAT Common Rare Results

IPA based pathway enrichment of the SKAT-O results revealed the lowest *p*-value and largest gene overlap for the α-tocopherol degradation pathway (*p*-value, 25% overlap), with the cytochrome P450 family 4 subfamily F member 2 (*CYP4F2*) gene being the main contributor (Supplementary Table 8, Supplementary Figure 2). Functional annotation of genes related to immune regulation, apoptosis, and autophagy again implicated *CYP4F2* in several functions, but otherwise yielded no relevant results (Supplementary Table 9). Finally, when investigating enrichment for genes involved in physiological system development the nervous system development had the lowest *p-*value, with the FERM, ARH/RhoGEF and Pleckstrin Domain Protein 2 (*FARP2*) gene driving this enrichment (Supplementary Table 10). While FARP2 has been implicated in CNS development, it is unlikely that *FARP2* plays a role in TBM pathogenesis as neurite remodeling is likely to affect neural connections and development (79). Additionally the Nucleotide binding and oligomerisation domain 2 (*NOD2/CARD15*) gene, involved in pathogen recognition and immune responses (80) and previously investigated as a TB candidate gene (81), was implicated in multiple developmental pathways, but the impact of this on TBM is unclear.

Pathway enrichment of the SKAT common rare analysis identified the S-methyl-5'-thioadenosine Degradation II pathway as the most significant, however as only one gene, methylthioadenosine phosphorylase (*MTAP*), was implicated in this pathway it was not investigated further (Supplementary Table 11). Enrichment of regulatory factors and control of the target genes identified several genes found to be under the control of common regulatory elements (Supplementary Table 12). *CD44* was found to target four genes in the input dataset, namely DNA (cytosine-5) methyltransferase 1 (*DNMT1*), interferon induced transmembrane protein 2 (*IFITM2*), interleukin 1 receptor associated kinase 3 (*IRAK3*) and programmed cell death 4 (*PDCD4*). Both *IFITM2* and *IRAK3* play important roles in immune signal transduction pathways. Enrichment for physiological system development again highlighted genes involved in neurological development among the top hits (Supplementary Table 13), with alpha 2 macroglobulin (*A2M*) being of particular interest. This result was relevant given that hits for the enrichment of genes involved in the proper development and functioning of neurological, embryonic, hematological and humoral immune response systems were all highlighted. This could point toward defects in the development and function of several critical physiological functions forming part of the etiology of TBM.

Network analysis showed that two of the input genes, receptor tyrosine kinase-like orphan receptor 1 (*ROR1*) and *DNMT1*, both regulate cadherin-1 (*CDH1*), a critical tight junction protein in the blood-brain barrier (BBB) (Supplementary Figure 3). *ROR1* was also identified in several functional enrichments (Supplementary Table 14), including cell morphology, cellular assembly and organization and cellular function and maintenance. *ROR1* may regulate other critical tight junction proteins at the BBB through the use of the transcription factor Snail-1 (*SNAI1*) (82).

Finally, the top hit genes were analyzed for possible involvement in pathways that have been previously implicated in TBM or pTB pathogenesis, for example immune system regulation and autophagy related processes. An additional 12 genes (Supplementary Table 15) were found that may play important roles in known TBM pathogenesis pathways but have yet to be investigated in the context of this disease.

## Discussion

### GWAS–TBM vs. Healthy Controls

Using logistic regression for SNP testing and correction for multiple testing and covariates, no significant genome-wide associations with TBM disease were detected. Three of the top four SNPs (rs3760495, rs2750007 and rs2273454) were in or around the Glyoxalase Domain Containing 4 (*GLOD4*) gene. In addition to this, all three displayed odds ratios pointing toward being susceptibility factors. Further investigation into the biological relevance of SNPs showed that rs3760495 and rs2750007 were intronic and rs2273454 was located upstream of *GLOD4* (Supplementary Table 2). All 3 of these SNPs were located in transcription factor binding sites bound by multiple transcription factors. Of particular interest was rs2273454 which was found to be in a binding site for RNA polymerase II. This points to the SNP being located in the *GLOD4* promoter region and may affect gene expression. As a result, future investigation into possible diminished RNA polymerase II binding and therefore diminished transcription of *GLOD4* as a result of rs2273454 should be investigated. Gene-based association testing (implemented in FUMA) based on the GWAS summary statistics revealed a significant association in a lncRNA with unknown implication in TBM susceptibility. While not reaching the Bonferroni significance threshold genes among the top hits are of potential interest for further investigation, including *GLOD4, PLEXNB2* and *MAPK11*. The function of *GLOD4* is poorly defined but it has been shown to interact with ADP ribosylation mechanisms, which is of interest here as the FUMA gene set analysis identified ADP ribosylation related genes to be significantly enriched for, based on the GWAS summary statistics (83, 84). As ADP ribosylation is involved in cell proliferation and differentiation and immune responses (78), *GLOD4* could influence TBM susceptibility by influencing ADP ribosylation. *MAPK11* and *PLEXNB2* have immune related implications and considering the implication of *MAPK* in pTB susceptibility these genes are of potential interest for future investigations (76, 77).

Considering the sample size used in the GWAS, the lack of genome-wide association is likely attributable to the diminished study power observed when using a study population of 123 TBM patients and 477 healthy controls. This does not imply that these SNPs may not be associated with TBM in other population groups because of allele frequency differences or when examined in a larger sample size with greater statistical power to detect associations.

### GWAS–TBM Cases vs. pTB Cases

The GWAS of TBM cases compared to pTB cases did not yield any SNPs that were associated with progression from pTB to TBM (Supplementary Table 4). A single exonic SNP, rs2251220, was amongst the top hits. Assessment of the substitution induced by rs2251220 using the prediction tools SiFT and Polyphen-2 determined that the non-synonymous change from a Serine to Leucine was benign and tolerated, respectively (85, 86). SNP selection for the MEGA array was based primarily around the selection of tag-SNPs therefore the location of the SNP being investigated is often not the true location of the SNP that is driving the effects seen in this study. Therefore, one cannot discount that these SNPs may be associated with TBM progression in other populations with different allele frequency spectrums. In that case, the LD structure surrounding each SNP would require investigation to determine the SNP truly driving the association and determine its biological relevance.

### Exome Sequencing

#### SKAT-O

Pathway enrichment analysis identified the *CYP4F2* gene (Supplementary Table 8, Supplementary Figure 2) as a significant part of the α-tocopherol degradation pathway (p_enrichment_ = 1x10^−3^). Further functional investigation of *CYP4F2* showed involvement in the early stages of leukotriene B4 (LTB4) degradation (75, 87, 88). This is of interest as the *LTA4H* locus has previously been shown to play a role in in TBM susceptibility due to its effects on TNF-α levels (40, 41). Specifically, the control of TNF-α concentrations was found to differ based on genotype. Wild-type homozygotes showed hyper-inflammation due to an excess of LTB4, which in turn led to an excessive TNF-α response. The involvement of *CYP4F2* in LTB4 regulation is mediated by degradation of LTB4 through ω-hydroxylation leading to the attenuation of TNF-α signaling thus modulating the inflammatory response along with the LTA4H enzyme (88). In addition, *CYP4F2* and *CYP4F3* have been shown to metabolize lipoxin A4 (LXA_4_) and lipoxin B4 (LXB_4_), both known anti-inflammatory lipoxins (89). An excess of LXA_4_ in particular has been shown to result in a hypo-inflammatory phenotype due to its anti-inflammatory actions on *TNF-*α. Therefore, a contributing factor to this excessive LTB_4_ and LXA_4_ could be attributed to non-synonymous SNPs in *CYP4F2* causing a loss of protein function, with rs3952537 predicted to be damaging by SiFT, Polyphen-2 and loss-of-function protein effect predictions as provided by the variant effect predictor (VEP) accessed through Ensembl (68).

Nucleotide binding and oligomerisation domain (*NOD2*) formerly known as *CARD15*, was found to be important in the development of three independent physiological systems including embryonic, hair and skin and renal developmental processes. *NOD2*, however, is of greatest interest for its role in innate immunity and is expressed on the surface of astrocytes and microglia in the CNS and functions as a recognition receptor for *S. pneumoniae* LPS derived muramyl dipeptide (90). Additionally, it has been implicated in the generation of IL-6 and TNF-α pro-inflammatory cytokines through the stimulation of NFκB toward other forms of bacterial meningitis caused by *N. meningitidis* and *B. bergdorferi* (80). Through the use of two murine models of *NOD2*^+/+^ and *NOD2*^−/−^, acute CNS inflammation was demonstrated with increased levels of CCL3, TNF-α and decreased levels of anti-inflammatory IL-10 in *NOD2*^+/+^ mice (90). This demonstrates a plausible role for *NOD2* in the generation of a detrimental inflammatory response as observed in TBM.

#### SKAT Common Rare

SKAT Common Rare analysis found a single significant gene-set (*CCP110*) after correction for gender and multiple testing. *CCP110* functions in cell-cycle maintenance, centrosomal duplication and is a regulator of ciliogenesis (91, 92), but the impact on TBM is unknown. Interestingly, in pneumococcal meningitis the loss of ciliary function of the ependyma contributes to the neuropathology observed (93).

The IPA analysis of the SKAT Common Rare top associations revealed six genes known to function in the development of the nervous system. This is critical in TBM pathogenesis as incomplete or poor development of protection mechanisms in crucial nervous system barriers such as the BBB, may expose the nervous system to pathogenic attack. One of the genes involved in nervous system development, alpha 2 macroglobulin (*A2M*), functions as a protease inhibitor for all four classes of proteases and is also a cytokine transporter for TNF-α, IL-6 and IL-1β amongst others (94). *A2M* along with IL-6 and C-Reactive-Protein (CRP) levels in the CSF can indicate blood-cerebrospinal fluid barrier (BCB) damage in bacterial meningitis as part of the acute phase reaction (95). This gene is also a possible biomarker for disease progression in both pTB and all forms of EPTB largely due to its role in IL-6 transport (96, 97). It is therefore a candidate for future studies as a potential biomarker and for functional studies in the context of TBM. Given the small sample size of the exome sequencing data, these findings should be considered as exploratory.

## Conclusion

This study contributes significantly to the TB host genetics field, as it presents the first exome sequencing study and GWAS of South African children with TBM, giving insight into manifestations of TBM patients in this setting. The overarching aim of this study was to assess the contribution of common and rare SNPs to TBM susceptibility.

GWAS and exome data analysis did not yield any strong significant results, likely due to the limited sample size (Table 1), stratification caused by the age difference between the TBM, healthy controls and pTB samples and the population stratification introduced by including both SAC and Xhosa individuals with different ethnic backgrounds and degrees of admixture. While we corrected for age in our analysis, future studies could focus on reducing the age stratification between cases and controls. Exploration of the GWAS and exome SKAT results led to the identification of numerous candidate genes (Supplementary Table 16) with implications in immune response and CNS development that could have a significant impact on TBM susceptibility and warrant further investigation. The pathogenic mechanisms leading to infection of the CNS and development of TBM are poorly understood and investigation of the proposed candidate genes could elucidate therapeutic targets to reduce the mortality rate of TBM patients.

TBM represents an extreme form of TB where the contribution of host genes and strain type are more likely to determine disease presentation than environmental factors (98). Our GWAS results support this hypothesis, since the TBM cases and pTB patients share similar environmental and economic circumstances. We also posit that defects during CNS development may contribute to TBM development rendering critical barriers such as the BBB or BCB vulnerable to pathogenic invasion. This is one of the largest TBM collections in Africa and therefore represents a valuable resource for TBM research.

## Data Availability Statement

The summary statistics from the case-control cohort will be made available to researchers on request, while access to the raw data will only be available to researchers who meet the criteria for access to confidential data after application to the Health Research Ethics Committee of Stellenbosch University. Requests can be sent to: MM, marlom@sun.ac.za.

## Ethics Statement

The studies involving human participants were reviewed and approved by the Health Research Ethics Committee of Stellenbosch University (Approval number 95/072 and N09/07/085). Written informed consent to participate in this study was provided by the participants or their legal guardian/next of kin.

## Author Contributions

RT, RS, and JS contributed to TBM sample collection and diagnosis. NB, BG, HS, CK, PH, EH, and MM made significant contributions to the analysis and interpretation of data. The work presented in the article was carried out in collaboration between all authors. All authors made substantial contributions to the conception or design of the work, critically revised the final manuscript, and read and approved the final manuscript.

## Funding

This research was partially funded by the South African Government through the South African Medical Research Council (SAMRC). Funding was also obtained from the South African National Research Foundation (Grant No: 91481 to MM) and Stellenbosch University.

## Author Disclaimer

The content is solely the responsibility of the authors and does not necessarily represent the official views of the SAMRC.

## Conflict of Interest

NB is currently an employee and shareholder of GlaxoSmithKline (Stevenage, UK).

The remaining authors declare that the research was conducted in the absence of any commercial or financial relationships that could be construed as a potential conflict of interest.

## Publisher's Note

All claims expressed in this article are solely those of the authors and do not necessarily represent those of their affiliated organizations, or those of the publisher, the editors and the reviewers. Any product that may be evaluated in this article, or claim that may be made by its manufacturer, is not guaranteed or endorsed by the publisher.
