# Supplementary material for: Deciphering Genetic Susceptibility to Tuberculous Meningitis
Source: Front Neurol. 2022 Mar 25;13:820168. doi: 10.3389/fneur.2022.820168 (PMC8993185; doi:10.3389/fneur.2022.820168)
Supplement: Supplementary file 1 [file Data_Sheet_1.docx]

**Supplementary Table 1:** Exploratory dataset for the TBM cases vs. healthy controls GWAS

| CHR^a^ | SNP | Base-pair Position | Unadjusted p-value | Genomic Control | Bonferroni Adjusted  p-value | Odds Ratio  [95% CI]^b^ |
| --- | --- | --- | --- | --- | --- | --- |
| 4 | rs77857429 | 22804085 | 5.68x10^-6^ | 7.17x10^-6^ | 1 | 4.28 [2.28-8.02] |
| 17 | rs3760495 | 684729 | 9.17x10^-6^ | 1.15x10^-5^ | 1 | 2.72 [1.75-4.24] |
| 17 | rs2273454 | 685640 | 9.17x10^-6^ | 1.15x10^-5^ | 1 | 2.72 [1.75-4.24] |
| 17 | rs2750007 | 677226 | 1.50x10^-5^ | 1.85x10^-5^ | 1 | 3.94 [2.12-7.33] |
| 3 | rs17046322 | 6952739 | 1.82x10^-5^ | 2.24x10^-5^ | 1 | 2.7 [1.71-4.25] |
| 4 | rs1511144 | 127535445 | 2.03x10^-5^ | 2.49x10^-5^ | 1 | 2.27 [1.56-3.3] |
| 10 | rs114255276 | 14825449 | 2.03x10^-5^ | 2.5x10^-5^ | 1 | 2.97 [1.8-4.89] |
| 18 | rs6565824 | 73487088 | 2.25x10^-5^ | 2.75x10^-5^ | 1 | 0.41 [0.27-0.62] |
| 22 | rs1127000 | 50716167 | 2.44x10^-5^ | 2.99x10^-5^ | 1 | 2.24 [1.54-3.26] |
| 12 | rs10774604 | 111168743 | 2.57x10^-5^ | 3.14x10^-5^ | 1 | 3.67 [2-6.73] |

***a. CHR = Chromosome***

***b. CI = Confidence Interval***

**Supplementary Table 2**: Localisation of each of the top hit SNPs from the GWAS: TBM cases vs. healthy controls

| SNP | Gene Symbol | Type of SNP | Transcription factor binding sites |
| --- | --- | --- | --- |
| rs77857429 | *GBA3* | Intronic | N/A |
| rs3760495 | *GLOD4* | Intronic | *CTCF, RAD21, SMC3* |
| rs2273454 | *GLOD4* | Upstream of gene | *POLR2A, SAP30, TCF3* |
| rs2750007 | *GLOD4* | Intronic | *FOXM1, RELA* |
| rs17046322 | *GRM7* | Intronic | N/A |
| rs1511144 | *LOC102724210/RBM48P1* | Intergenic | N/A |
| rs114255276 | *FAM107B* | Promoter Flanking | *GTF3C2* |
| rs6565824 | *LOC100505853* | Promoter Flanking | N/A |
| rs1127000 | *PLXNB2* | Splice site | N/A |
| rs10774604 | *PPP1CC* | Intronic | N/A |

**Supplementary Table 3:** Association statistics for previous TB and TBM candidate genes for the TBM vs. Healthy Controls analysis.

| Top hits for TB candidate SNPs and Genes | | | | | | |
| --- | --- | --- | --- | --- | --- | --- |
| SNP | **CHR** | **Base-pair**  **Position** | **A1** | **Odds Ratio [95% CI]** | **Unadjusted  p-value** | **Gene** |
| rs2115819 | 10 | 45901089 | G | 1.71 [1.20-2.44] | 0.00278 | *ALOX5* |
| rs12208808 | 6 | 163375679 | T | 3.80 [1.56-9.30] | 0.00338 | *PACRG* |
| rs2839695 | 10 | 44873849 | C | 0.40 [0.21-0.76] | 0.00496 | *CXCL12* |
| rs2844519 | 6 | 31371430 | A | 1.78 [1.17-2.71] | 0.00699 | *MICA* |
| rs9376267 | 6 | 137531031 | T | 0.51 [0.31-0.84] | 0.00780 | *IFNGR1* |
| rs12264801 | 10 | 45914744 | A | 1.60 [1.13-2.27] | 0.00818 | *ALOX5* |
| rs11968578 | 6 | 162202943 | G | 0.65 [0.46-0.91] | 0.0125 | *PRKN* |
| rs76209835 | 6 | 162976350 | C | 4.41 [1.31-14.82] | 0.0163 | *PRKN* |
| rs12202396 | 6 | 162597463 | C | 1.57 [1.08-2.29] | 0.0175 | *PRKN* |
| rs2235023 | 7 | 87190452 | A | 1.55 [1.08-2.23] | 0.0176 | *ABCB1* |
| rs2540499 | 12 | 96399869 | T | 0.55 [0.34-0.91] | 0.0177 | *LTA4H* |
| Top hits for TBM candidate Genes | | | | | | |
| SNP | **CHR**^a^ | **Base-pair Position** | **A1** | **Odds Ratio [95% CI]** ^b^ | **Unadjusted  p-value** | **Gene** |
| rs2540499 | 12 | 96399869 | T | 0.55 [0.25-0.34] | 0.0177 | *LTA4H* |
| rs1540339 | 12 | 48257326 | A | 0.65 [0.43-0.98] | 0.0373 | *VDR* |
| rs17025033 | 12 | 96400460 | T | 1.92 [1.02-3.63] | 0.0434 | *LTA4H* |
| rs11108384 | 12 | 96430674 | T | 0.27 [0.07-0.98] | 0.0464 | *LTA4H* |
| rs34153004 | 7 | 98720395 | G | 0.54 [0.29-1.02] | 0.0585 | *SMURF1* |
| Previously associated Candidate TBM SNPs available in our data | | | | | | |
| SNP | **CHR** | **Base-pair Position** | **A1** | **Odds Ratio [95% CI]** | **Unadjusted  p-value** | **Gene** |
| rs731236 | 12 | 48238757 | G | 1.07 [0.73-1.58] | 0.732 | *VDR* |
| rs2072069 | 17 | 32684991 | A | 0.90 [0.63-1.29] | 0.571 | *CCL13* |

***a. CHR = Chromosome***

***b. CI = Confidence Interval***

**Supplementary Table 4:** Exploratory dataset for the TBM cases vs. pTB cases GWAS.

| Chr^a^ | Base-pair Position | SNP | Unadjusted p-value | Genomic  Control | Bonferroni Adjusted p-value | Odds Ratio  [95% CI]^b^ |
| --- | --- | --- | --- | --- | --- | --- |
| 16 | 80350711 | rs2679308 | 5.11x10^-6^ | 7.57x10^-6^ | 1 | 26.43 [6.47-108] |
| 17 | 51638548 | rs4309447 | 5.72x10^-6^ | 8.44x10^-6^ | 1 | 3.70 [2.10-6.50] |
| 2 | 121302719 | rs2140779 | 5.95x10^-6^ | 8.78x10^-6^ | 1 | 18.12 [5.17-63.5] |
| 17 | 51653566 | rs2055478 | 8.29x10^-6^ | 1.21x10^-5^ | 1 | 3.61 [2.05-6.34] |
| 5 | 1442842 | rs13189021 | 9.74x10^-6^ | 1.41x10^-5^ | 1 | 11.66 [3.93-34.61] |
| 7 | 138601826 | rs2251220 | 1.31x10^-5^ | 1.89x10^-5^ | 1 | 3.66 [2.04-6.57] |
| 13 | 112048353 | rs61966912 | 1.85x10^-5^ | 2.61x10^-5^ | 1 | 19.4 [4.99-75.35] |
| 4 | 166976065 | rs1393854 | 1.93x10^-5^ | 2.72x10^-5^ | 1 | 8.61 [3.21-23.11] |
| 4 | 168490955 | rs1914621 | 1.97x10^-5^ | 2.79x10^-5^ | 1 | 0.33 [0.20-0.55] |
| 1 | 168987665 | rs61803268 | 2.02x10^-5^ | 2.85x10^-5^ | 1 | 8.45 [3.17-22.54] |

***a. CHR = Chromosome***

***b. CI = Confidence Interval***

**Supplementary Table 5:** Localisation of each of the top-hit SNPs from the GWAS of TBM cases vs. pTB cases

| SNP | Gene | SNP Type | Transcription Factor Binding Sites |
| --- | --- | --- | --- |
| rs2679308 | *LOC102724084* | Intronic | N/A |
| rs4309447 | *LOC100419014/RPS2P48* | Intergenic | *FOS, JUND* |
| rs2140779 | *LOC84931/GLI2* | Upstream SNP | *JUND* |
| rs2055478 | *LOC100419014/RPS2P48* | Intergenic | N/A |
| rs13189021 | *SLC6A3* | Intronic | N/A |
| rs2251220 | *KIAA1549* | Exonic | N/A |
| rs61966912 | *TEX29/LOC101928616* | Intergenic | N/A |
| rs1393854 | *TLL1* | Intronic | N/A |
| rs1914621 | *SPOCK3/PHBP14* | Intergenic | N/A |
| rs61803268 | *LINC00970* | Intronic | N/A |

**Supplementary Table 6:** Association statistics for previous TB and TBM candidate genes for the TBM vs. pTB analysis.

| Top hits for TB candidate SNPs and Genes | | | | | | |
| --- | --- | --- | --- | --- | --- | --- |
| SNP | **CHR** | **Base-pair Position** | **A1** | **Odds Ratio [95% CI]** | **Unadjusted  p-value** | **Gene** |
| rs163787 | 20 | 57574463 | A | 3.38 [1.67-6.85] | 0.000698 | *CTSZ* |
| rs7772285 | 6 | 163658221 | T | 2.19 [1.36-3.55] | 0.00129 | *PACRG* |
| rs2741126 | 8 | 6731280 | C | 2.17 [1.34-3.54] | 0.00164 | *DEFB1* |
| rs305069 | 16 | 85949558 | T | 2.53 [1.53-4.73] | 0.00372 | *IRF8* |
| rs2069830 | 7 | 22767137 | T | 3.40 [1.35-8.55] | 0.00923 | *IL6* |
| rs10210465 | 2 | 231039578 | A | 2.73 [1.28-5.81] | 0.00932 | *SP110* |
| Top hits for TBM candidate Genes | | | | | | |
| SNP | **CHR** | **Base-pair Position** | **A1** | **Odds Ratio [95% CI]** | **Unadjusted  p-value** | **Gene** |
| rs11168284 | 12 | 48283049 | G | 0.57 [0.35-0.91] | 0.0189 | *VDR* |
| rs2853564 | 12 | 48278487 | C | 2.16 [1.10-4.23] | 0.0245 | *VDR* |
| rs1544410 | 12 | 48239835 | A | 1.78 [1.01-3.13] | 0.0442 | *VDR* |
| rs6465737 | 7 | 98725348 | G | 0.57 [0.33-0.99] | 0.0497 | *SMURF1* |

**Supplementary Table 7**: FUMA Gene-based association statistics based on the TBM vs. healthy control GWAS summary statistics.

| GENE | CHR | START (bp) | STOP (bp) | NSNPS | N sample | P-value |
| --- | --- | --- | --- | --- | --- | --- |
| *AL022328.1* | 22 | 50704103 | 50724350 | 10 | 505 | 2.06 x10^-6^ |
| *GLOD4* | 17 | 650337 | 695581 | 9 | 509 | 8.97 x10^-6^ |
| *MAPK11* | 22 | 50692142 | 50719196 | 13 | 504 | 1.39 x10^-5^ |
| *RNMTL1* | 17 | 675513 | 705749 | 10 | 508 | 2.15 x10^-5^ |
| *PLXNB2* | 22 | 50703408 | 50756056 | 20 | 504 | 2.44 x10^-5^ |
| *LSM14B* | 20 | 60687517 | 60720434 | 5 | 508 | 9.54 x10^-5^ |


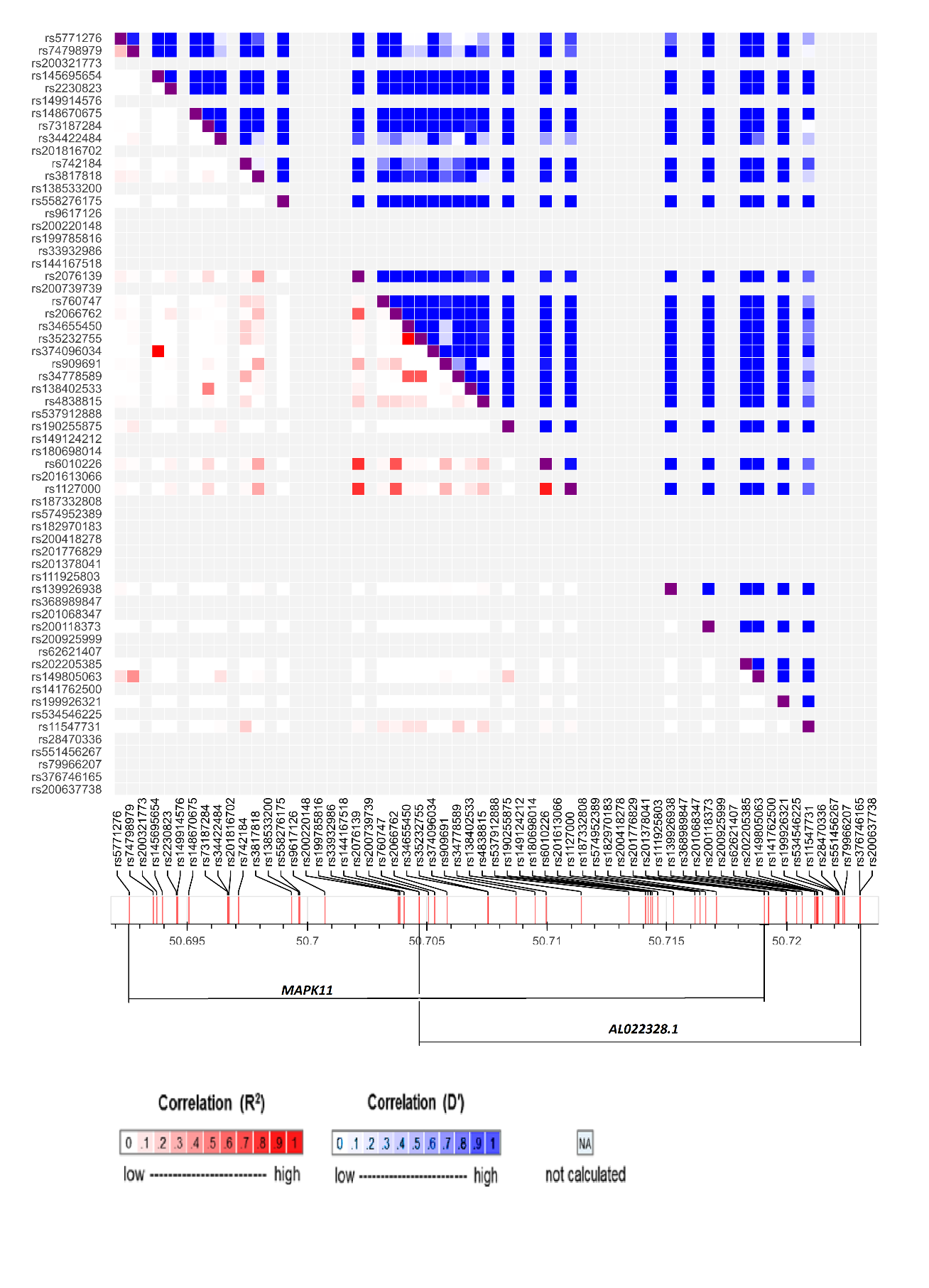


**Supplementary Figure 1:** Heat map showing the overlap and linkage disequilibrium between *MAPK11* (*mitogen-activated protein kinase 11*) and *AL022328.1.*

**Supplementary Table 8:** Highlighted canonical pathways in the SKAT-O analysis.

| Pathway Name | p-value | Overlap between input and total genes in pathway (Ratio) | Overlapping Genes |
| --- | --- | --- | --- |
| Transcriptional regulation network in embryonic stem cells | 2.90x10^-3^ | 5%, (2/40) | *RIF1, ZFHX3* |
| α-tocopherol Degradation | 7.98x10^-3^ | 25% (1/4) | *CYP4F2* |
| Histamine Degradation | 2.57x10^-2^ | 7.7% (1/13) | *ALDH3B1* |
| Oxidative Ethanol Degradation III | 2.96x10^-2^ | 6.7% (1/15) | *ALDH3B1* |
| Fatty Acid α-oxidation | 3.15x10^-2^ | 6.2% (1/16) | *ALDH3B1* |


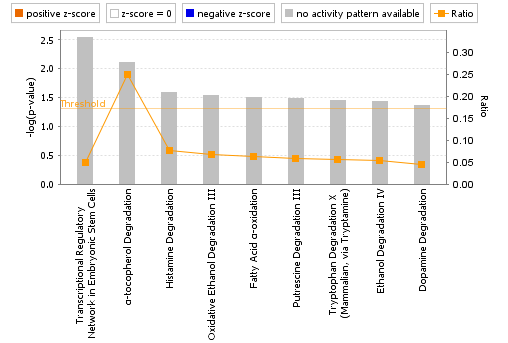


**Supplementary Figure 2:** Canonical pathways highlighted in IPA analysis

**Supplementary Table 9:** Enrichments for molecular function in the SKAT-O analysis.

| Functional Annotation | p-value range | Annotated Genes |
| --- | --- | --- |
| Lipid Metabolism | 3.35x10^-2^ – 2x10^-3^ | *CYP4F2, PIGG, GCKR* |
| Small Molecule Biochemistry | 3.35x10^-2^ – 2x10^-3^ | *CYP4F2, ALDH3B1, PIGG, GCKR, ITPR3, ESRRA* |
| Vitamin and Mineral Metabolism | 3.15x10^-2^ – 2x10^-3^ | *CYP4F2, ITPR3* |
| Cell Death and Survival | 4x10^-3^ | *PHC1* |
| Cellular Assembly and Organisation | 2.77x10^-2^ – 4x10^-3^ | *FARP2, TOPBP1, ESPL1* |

**Supplementary Table 10:** The development and function of physiological systems most affected by genes highlighted by the SKAT-O analysis.

| Physiological System | p-value range | Annotated Genes |
| --- | --- | --- |
| Nervous System development | 4x10^-3^ | *FARP2* |
| Embryonic Development | 3.15x10^-2^ – 4.85x10^-3^ | *CC2D1A, NOD2* |
| Hair and Skin Development | 9.96x10^-3^ – 4.85x10^-3^ | *CC2D1A, NOD2* |
| Renal and Urological Development | 9.96x10^-3^ – 5.22x10^-3^ | *CC2D1A, NOD2* |
| Skeletal and Muscular Development | 4.89x10^-2^ | *ZFHX3* |

**Supplementary Table 11:** Canonical pathways highlighted in IPA analysis.

| Pathway Name | p-value | Overlap of input genes with known pathways | Genes Associated |
| --- | --- | --- | --- |
| S-methyl-5'-thioadenosine Degradation II | 7.53x10^-3^ | 100%, (1/1) | *MTAP* |
| Glycine Biosynthesis III | 1.50x10^-2^ | 50%, (1/2) | *AGXT2* |
| Role of Oct4 in Mammalian Embryonic Stem Cell Pluripotency | 4.51x10^-2^ | 4.4 %, (2/45) | *FAM208A, NR5A1* |
| Phototransduction Pathway | 5.65x10^-2^ | 3.9 %, (2/51) | *GNAT2, GUCA1C* |
| FXR/RXR Activation | 6.85x10^-2^ | 2.4 %, (3/125) | *APOF, G6PC, G6PC3* |

**Supplementary Table 12:** Common regulatory elements for the top-hits of SKAT Common/Rare.

| Regulatory Element | p-value |
| --- | --- |
| CD44 | 3.10x10^-4^ |
| PSMD4 | 5.11x10^-4^ |
| PPP2CB | 7.22x10^-3^ |
| TIPIN | 7.22x10^-3^ |
| TAL1 | 1.21x10^-2^ |

**Supplementary Table 13:** Enrichment of genes that correspond to the development and function of physiological systems.

| Physiological system development and function | p-value | Genes ascribed to pathway |
| --- | --- | --- |
| Nervous System | 3.71x10^-2^ - 1.68x10^-4^ | *A2M, CX3CR1, CYFIP1, GNRH1, MACF1, NLGN4X* |
| Embryonic Development | 3.71x10^-2^ - 7.53x10^-3^ | *GNRH1, NR5A1, NLGN4X* |
| Hair and Skin | 7.53x10^-3^ | *KRT20* |
| Haematological system | 4.43x10^-2^ - 7.53x10^-3^ | *CX3CR1, MARCO, A2M, PNPT1, HAVCR1, CLC* |
| Humoral Immune Response | 7.53x10^-3^ | *HAVCR1* |


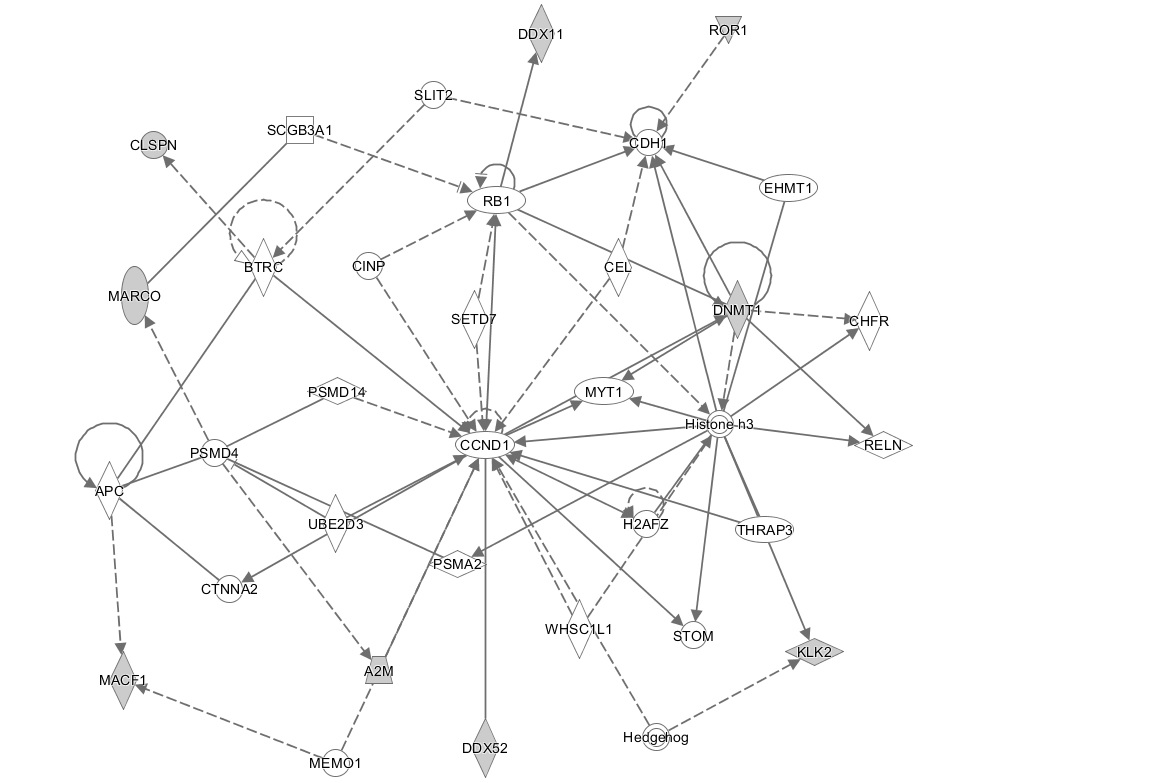


**Supplementary Figure 3:** The relationships between CDH1, ROR1 and DMNT1. ROR1 interacts indirectly through other proteins to exact its regulatory function upon CDH1, denoted by the broken line. DNMT1 interacts with CDH1 in a direct protein-protein interaction, denoted by the solid line.

**Supplementary Table 14:** Enrichments for molecular and cellular functions.

| Functional Annotation | p-value | Top-Hit Genes Associated |
| --- | --- | --- |
| Cellular Movement | 4.43x10^-2^ - 1.68x10^-4^ | *A2M, GNRH1, MYO7A* |
| Cell Morphology | 4.43x10^-2^ - 4.42x10^-3^ | *CYFIP1, MACF1, ROR1, ZFYVE16* |
| Cellular Assembly and Organisation | 4.43x10^-2^ - 4.42x10^-3^ | *CYFIP1, MACF1, ROR1, KRT20, MAP2, PACS2, EXO5, ANLN, DDX11, ZFYVE16, SPAG16, NLGN4X, MYO7A* |
| Cellular Function and Maintenance | 4.43x10^-2^ - 4.42x10^-3^ | *CYFIP1, MACF1, ROR1, KRT20, A2M, SPAG16, MAP2, NLGN4X, MYO7A* |
| Cell Cycle | 2.98x10^-2^ - 7.53x10^-3^ | *PNTP1, HAVCR1, EXO5, CLSPN* |

**Supplementary Table 15:** Genes from the SKAT Common/Rare top hits that function in pathways thought to be involved in TBM pathogenesis.

| Gene ID | Gene Name | Function and Pathway |
| --- | --- | --- |
| *A2M* | Alpha 2 Macroglobulin | Cytokine transporter, IL-6 pathway |
| *ATG10* | Autophagy-related 10 | Autophagy – Autophagosome formation |
| *C4BPA* | Complement component 4 binding protein, alpha | Regulation of the complement system |
| *CX3CR1* | Chemokine (C-X3-C motif) receptor 1 | Leukocyte adhesion and migration, activation of microglia |
| *IRAK3* | Interleukin 1 receptor associated kinase 3 | Component of the toll/IL-1 pathways, negative regulator of TLR signalling |
| *MARCO* | Macrophage receptor with collagenous structure | Innate immune signalling, Recognition receptor |
| *IFITM2* | Interferon induced transmembrane protein 2 | Restriction of *M.tb* infection, Phagosome maturation |

**Supplementary Table 16:** Potential TBM candidate genes identified in this study.

| Gene | Association Test | Unadjusted p-value | Relevance |
| --- | --- | --- | --- |
| *ZFHX3* | SKAT-O | 4.63x10^-4^ | SKAT-O, Lowest p-value |
| *CYP4F2* | SKAT-O | 1x10^-3^ | LTB_4_ regulation |
| *NOD2* | SKAT-O | 6.71x10^-3^ | TNF-α regulation and pathogen recognition |
| ***CCP110*** | **SKAT Common Rare** | **5.89x10^-6^** | **SKAT Common Rare lowest p-value** |
| *A2M* | SKAT Common Rare | 8.63x10^-3^ | Cytokine transporter |
| *CX3CR1* | SKAT Common Rare | 9.57x10^-3^ | T and B cell recruitment, microglia response |
| *MARCO* | SKAT Common Rare | 1.75x10^-3^ | Pathogen recognition |
| *ROR1* | SKAT Common Rare | 6.60x10^-4^ | Regulation of tight junctions |
| *ATG10* | SKAT Common Rare | 8.56x10^-4^ | Autophagy – phagosomal maturation |
| *IFITM2* | SKAT Common Rare | 9.23x10^-3^ | Innate immunity – signal transduction |
| *C4BPA* | SKAT Common Rare | 2.54x10^-4^ | Complement system regulation |
| *IRAK-M* | SKAT Common Rare | 8.24x10^-3^ | TLR pathway regulation |

*Significant results are highlighted in bold text*
